# Supplementary material for: A high-resolution mRNA expression time course of embryonic development in zebrafish
Source: eLife. 2017 Nov 16;6:e30860. doi: 10.7554/eLife.30860 (PMC5690287; doi:10.7554/eLife.30860)
Supplement: Supplementary file 6. [file elife-30860-supp6.zip › biolayout-clusters-files/Cluster246-genes.html]

Cluster246


# Cluster246: Genes

| Ensembl ID | Gene Name | Chr | Start | End | Biotype |
| --- | --- | --- | --- | --- | --- |
| ENSDARG00000071774 | eef1e1 | 24 | 8199506 | 8217207 | protein\_coding |
| ENSDARG00000086618 | psma3 | 17 | 11267577 | 11285749 | protein\_coding |
| ENSDARG00000005134 | psmd11b | 12 | 28234935 | 28248209 | protein\_coding |
| ENSDARG00000052377 | psmd12 | 6 | 19465326 | 19474013 | protein\_coding |
| ENSDARG00000058220 | tada3l | 11 | 16017681 | 16031405 | protein\_coding |
| ENSDARG00000020008 | vcp | 5 | 40910234 | 40920827 | protein\_coding |
